# Developing a culturally informed telepsychiatry competency framework for aotearoa New Zealand: A cross-sectional survey and factor analysis

**DOI:** 10.1177/10398562251345313

**Published:** 2025-05-28

**Authors:** Alisha Vara, Yan Chen, Etuini Ma’u, Rodrigo Ramalho, Mark Lawrence, Gary Cheung

**Affiliations:** Department of Psychological Medicine, Faculty of Medical and Health Sciences, 62710 The University of Auckland, Auckland, New Zealand; Centre for Medical and Health Sciences Education, Faculty of Medical and Health Sciences, 62710 The University of Auckland, Auckland, New Zealand; Department of Psychological Medicine, Faculty of Medical and Health Sciences, 62710 The University of Auckland, Auckland, New Zealand; Department of Social and Community Health, Faculty of Medical and Health Sciences, 62710 The University of Auckland, Auckland, New Zealand; Mental Health and Addiction Services, 7854 Health New Zealand Hauora a Toi Bay of Plenty, Tauranga, New Zealand; Department of Psychological Medicine, Faculty of Medical and Health Sciences, 62710 The University of Auckland, Auckland, New Zealand

**Keywords:** telepsychiatry, competency, culture, mental health services, videoconferencing

## Abstract

**Objective:**

The COVID-19 pandemic accelerated telepsychiatry use in New Zealand. This study aimed to survey New Zealand psychiatrists and trainees’ perception of their telepsychiatry competencies; the importance of these competencies when providing a culturally responsive telepsychiatry service; and to ascertain if the competency items converged to dimensions developed from a previous qualitative study.

**Methods:**

New Zealand psychiatrists and trainees completed an anonymised online questionnaire comprising 20 competency statements. Participants rated each statement on a scale from 0 (low level competence/importance) to 100 (high level competence/importance): (1) How competent do you believe you are? (2) How important is this competency when providing a culturally responsive telepsychiatry service? A principal components factor analysis with Varimax Rotation was conducted on the importance ratings.

**Results:**

Eighty-six participants (47.7% female) participated. The factor analysis revealed four new domains: Cultural Safety, Infrastructure and Technology, Professional Development and Ethics, and Clinical Practice. These new domains explained 64.9% of the total variance. Their internal consistencies were acceptable (alpha≥0.70) except for self-rated competence in the Clinical Practice domain (alpha = 0.68).

**Conclusion:**

This competency framework could be used to develop educational curricula and training resources to improve culturally responsive telepsychiatry practice in Aotearoa.

Telepsychiatry is defined as ‘the practice of telehealth to provide mental health care, which is a live, real-time, synchronous, two-way videoconferencing communication on the receiving and delivery end’.^1^ Historically, telepsychiatry was under-utilised, and there was a lack of guidance in developing educational curricula for its use.^2,3^ In Aotearoa New Zealand, the government-mandated COVID-19 lockdowns accelerated the use of telepsychiatry to continue provision of psychiatric services when face-to-face interactions were not possible. Since the COVID-19 pandemic, there has been an emergence of interest and guidance in using telepsychiatry as a complementary and valuable tool alongside in-person service delivery.^4^

Public health services in Aotearoa have an obligation to address mental health inequities^5^ and require health care systems and providers to engage in working towards cultural safety.^6^ Cultural safety requires individuals and organisations including professional training bodies in healthcare to consider the impact of their own culture on healthcare service delivery and to take accountability for providing culturally safe care for patients, to achieve health equity.^6^ Māori, the Indigenous people of Aotearoa, and Pacific peoples both have a higher burden of psychiatric disorders than other ethnic groups.^7^ Health systems that improve access for vulnerable groups should be safe and accessible. Successful cultural adaptation of telepsychiatry services for Indigenous populations and ethnic minorities requires involvement of these communities alongside quality service delivery to increase access and engagement.^8^ Barriers to cultural adaptation identified in the literature include those related to health service and infrastructure, and to service users’ socioeconomic and cultural contexts.^8^ Planning telepsychiatry services to deliver care to these, and all other communities, requires collaborative development of standards, regulations, and guidelines with all stakeholders to promote culturally safe service delivery^8,9^ to maximise positive outcomes and address inequities.

Qualitative research investigating mental health clinicians’ telehealth experiences in Aotearoa during the COVID-19 pandemic^10^ found that the rapid change in service delivery, including the move to telehealth, caused uncertainty with lack of guidance in setting up telehealth workplaces and decreased quality of interpersonal client relationships. Telehealth did bring advantages for some clinicians such as convenience to attend supervision and increased accessibility to colleagues. Māori clinicians were especially impacted by isolation given the value that Māori place on physical connections and described that continued practice of culture-specific activities such as *karakia* (prayers or incantation) supported team well-being.^10^ The need for cultural safety and consultation with Māori stakeholders for better application of telehealth were emphasised. Additional research exploring the views of mental health clients engaged in telehealth during the COVID-19 pandemic in Aotearoa^11^ revealed practical considerations such as setting parameters for telehealth appointments and providing appropriate training and resources for clinicians to comfortably and safely conduct telehealth appointments and assessments.

Our research team previously used qualitative methodology to explore psychiatrist and trainee experiences of telepsychiatry during the COVID-19 pandemic.^12^ Psychiatrists and trainees reflected that cultural safety was integral to clinical practice and service delivery in a telepsychiatry environment. Telepsychiatry was also found to be advantageous when including *whānau* (family members), especially for Māori, Pacific and Asian clients and additionally using interpreters. Conversely there were concerns for barriers to accessing technology for Māori and Pacific and less ability to build rapport with the loss of *kanohi ki te kanohi* (face to face) interactions with clients. However, telepsychiatry posed challenges to clinical practice including continuity of care, patient selection, and psychiatric examination. Factors such as flexible working conditions positively contributed to their experience, while lack of technical expertise and training detracted from their competency in telepsychiatry. The need for formal training and guidance in particular related to cultural safety, risk assessment, and privacy concerns in telepsychiatry was highlighted.

Although there is emerging evidence in Aotearoa about the experiences of psychiatrists and trainees with telepsychiatry, there is a lack of consensus regarding specific competencies for effective and culturally safe delivery of telepsychiatry. Previous research has identified telepsychiatry training competencies for psychiatry residents through a qualitative grounded theory approach with guidance from the literature.^13^ Following our aforementioned qualitative study,^12^ the research team, which included three academic psychiatrists with expertise in telepsychiatry, a Māori psychiatrist, a Pacific psychiatrist, two psychiatry trainees and a medical educationist worked to develop culturally informed telepsychiatry competencies. First, proposed competencies were extracted from the qualitative study raw data. These competencies were reviewed against the Royal Australian & New Zealand College of Psychiatrists Professional Practice Guideline for telehealth in psychiatry^14^ and a systematic scoping review and thematic analysis identifying factors to guide the adaptation of telepsychiatry to meet the needs of Indigenous peoples and racial and ethnic minorities.^8^ Lastly, the research team met to develop consensus on the final competencies used in this study (see Supplemental Material).

This study aimed to survey psychiatrists and trainees’ perception of their competencies and secondly their opinion on the importance of these competencies when providing a culturally responsive telepsychiatry service. A third aim was to ascertain if the competency items converged to dimensions similar to those developed from our previous qualitative study.^12^ The results of this study can inform the development of a cultural competency framework to guide telepsychiatry practice, including the provision of education and guidelines to deliver telepsychiatry in Aotearoa effectively.

## Methods

### Participants and setting

Following approval by the University of Auckland Human Participants Ethics Committee, the Royal Australian and New Zealand College of Psychiatrists (RANZCP) New Zealand National Office invited all New Zealand psychiatrists (*n* = 608) and trainees (*n* = 240) via email to participate in an online questionnaire administered using the QualtricsXM software. The survey took place in May 2021, with a reminder sent to all potential participants after two weeks.

### Data collection and survey questions

Anonymised demographic data were collected. We randomly ordered our proposed 20 competency statements required for practising telepsychiatry in Aotearoa (see Supplemental Material). These 20 competency statements were preliminarily grouped in four domains (Cultural Safety, Infrastructure and Technology, Professionalism and Practice, Special Populations and Clinical situations); they were later refined after a principal component factor (PCF) analysis described in the section on data analysis. For each of the 20 competency statements, we asked the participants to rate two questions on a Visual Analogue Scale from 0 (low level of competence/importance) to 100 (high level of competence/importance). These questions were:

(1) How competent do you believe you are?
(2) How important is this competency when providing a culturally responsive telepsychiatry service?

### Data analysis

Descriptive statistics were performed to describe the participants’ demographics (*n*, %). Chi-squared tests were used to determine whether there was any statistically significant difference (*p* < .05) in the demographics between participants who completed all the survey questions and those who partially completed the questions. We calculated the means and standard deviations of participants’ ratings of the two survey questions (Self-rated Competence and Importance) across the 20 competency statements. The Importance-Competence Gap for each statement was determined by subtracting the mean Self-rated Competence from the mean Importance rating. Next, we conducted a PCF analysis with Varimax Rotation on the importance ratings, which included the total variance explained by the four preliminary domains (factors). We chose the importance rating to capture participants’ perceptions of a competency framework. Competency statements that were loaded on two or more domains (factors) in the PCF analysis were eliminated to form the final competency framework. Finally, we reported internal consistency, measured by Cronbach’s alpha, for each of the new competency domains (factors). An alpha of ≥0.70 suggests an acceptable level of internal consistency.^15^

## Results

Eighty-six participants (psychiatrists *n* = 70, 81.4% and trainees *n* = 16, 18.6%) consented to participate. The response rates were 11.5% for psychiatrists and 6.7% for trainees. Most participants (*n* = 29, 33.7%) were aged 60 and over; the remaining participants were aged 50–59 (*n* = 27, 31.4%), 40-49 (*n* = 13, 15.1%), 30–39 (*n* = 11, 12.8%), and 20–29 (*n* = 6, 7.0%). There were similar proportions of males (*n* = 42, 48.8%) and females (*n* = 41, 47.7%), and three participants (3.5%) identified as gender diverse. Most participants were New Zealand European (*n* = 52, 60.5%), followed by Asian (*n* = 7, 8.1%), Māori (*n* = 2, 2.3%), Pacific (*n* = 2.3%) and Other (*n* = 27, 31.4%). Eighteen participants partially completed the survey. There were no significant differences in age group, ethnicity or professional group between those who completed the entire survey versus those who partially completed it. However, the group that completed the full survey had statistically significantly more males (*n* = 37, 43.0%) than the group that partially completed it (*n* = 5, 5.8%). Remaining analyses were conducted with all participants, regardless of whether they completed the entire survey.

The results of the descriptive analysis of the 20 competency statements are summarised in Table 1. The three statements scoring the lowest in Self-rated Competence were: #1 ‘Applies official guidelines on cultural safety’; #5 ‘Can work effectively with an interpreter’; #8 ‘Can address technological problems and issues that may arise for a patient’. The three competency statements scoring the highest in Importance when providing a culturally responsive telepsychiatry service were: #16 ‘Can build rapport and trust and show empathy with a patient’; #18 ‘Recognises the relative contraindications and limitations of using telepsychiatry’; #19 ‘Can manage patient’s risks to self or others when they arise during a telepsychiatry consultation’. The three competency statements that have the largest Importance-Competence gap were: #1, #5 and #19.

**Table 1.** Means and standard deviations of self-rated competency and importance of the 20 competency statements by 86 psychiatrists and trainees (0 = low level of competence/importance, 100 = high level of competence/importance).

Competency statement	*N* ^a^	Self-rated competence Mean (SD)	Importance Mean (SD)
Domain 1: Cultural safety			
1	74	56.88 (24.19)	78.49 (18.59)
2	71	76.11 (18.49)	83.94 (16.36)
3	69	70.61 (23.41)	83.86 (15.15)
4	70	74.43 (21.55)	86.07 (15.76)
5	73	62.08 (23.13)	81.48 (14.30)
Domain 2: Infrastructure and technology			
6	69	67.67 (24.39)	75.30 (17.64)
7	70	70.17 (21.91)	81.27 (14.81)
8	70	60.74 (26.61)	80.64 (13.82)
9	70	62.41 (26.15)	78.07 (13.70)
Domain 3: Professionalism and practice			
10	70	70.79 (23.21)	78.41 (17.98)
11	70	65.43 (26.94)	77.66 (16.41)
12	73	86.26 (15.32)	86.90 (14.53)
13	71	66.73 (24.49)	85.65 (12.94)
14	72	73.61 (19.47)	79.86 (16.03)
15	68	71.56 (19.15)	79.24 (19.03)
Domain 4: Special populations and clinical situations			
16	70	77.74 (18.02)	88.94 (9.84)
17	69	73.17 (18.16)	79.64 (15.46)
18	72	78.06 (17.42)	87.18 (11.46)
19	70	67.70 (21.64)	89.66 (11.19)
20	73	69.15 (23.62)	86.05 (16.55)

^a^18 participants did not complete the two questions for all 20 competency statements: (1) How competent do you believe you are? (2) How important is this competency when?

### PCF analysis

The preliminary PCF analysis with Varimax Rotation on the importance ratings revealed a four-factor solution explaining 64.9% of the total variance (see Table 2 for a summary of the total variance explained by the four factors). The rotated component matrix showed that two competency statements (#7 & #19) loaded on two factors, and they were eliminated in the main PCF analysis. #19 ‘Can manage patient’s risks to self or others when they arise during a telepsychiatry consultation’ was one of the three competencies that was highly rated by the survey participants in terms of its importance when providing a culturally responsive telepsychiatry service. Although #19 was eliminated in the final questionnaire, #18 ‘Recognises the relative contraindications and limitations of using telepsychiatry in certain clinical populations and situations’ already covered an example of patients who present with significant risk and safety issues. The new factor structure, item loadings, and revised competency domain names are shown in Table 3. The PCF analysis revealed four domains: Cultural safety, Infrastructure and Technology, Professional Development and Ethics, and Clinical Practice. The final four competency domains and 18 competency statements are shown in Table 4. The four new domains’ internal consistencies were acceptable (alpha≥0.70), except for self-rated competence in the Clinical Practice domain (alpha = 0.68) (Table 5).

**Table 2.** Total variance explained by the preliminary principal components factor analysis.

	Total	% Of variance	Cumulative %
Factor 1	8.61	43.05	43.05
Factor 2	1.87	9.34	52.38
Factor 3	1.33	6.66	59.04
Factor 4	1.16	5.82	61.86

**Table 3.** Factor structure and item loadings for 18 competency statements in the main principal components factor analysis.

	Factor 1: Cultural safety	Factor 2: Infrastructure and technology	Factor 3: Professional development and ethics	Factor 4: Clinical practice
Item #1	0.69			
Item #2	0.62			
Item #3	0.80			
Item #4	0.76			
Item #5	0.58			
Item #16	0.55			
Item #6		0.80		
Item #8		0.80		
Item #9		0.60		
Item #17		0.70		
Item #10			0.75	
Item #11			0.76	
Item #13			0.71	
Item #15			0.66	
Item #12				0.81
Item #14				0.59
Item #18				0.57
Item #20				0.65

**Table 4.** The final four competency domains and 18 competency statements.

Domain 1: Cultural safety NB: Domain 1 competency statements form the foundation for competency statements listed in the other 3 domains	
1)	Applies official guidelines on cultural safety when practising telepsychiatry. For example, the New Zealand medical Council’s statement on cultural safety and He ara hauora māori: A pathway to māori health equity
2)	Demonstrates an appreciation of inequity in accessing telepsychiatry in some communities and endeavours to accommodate these. For example, practical issues and wider social considerations of a patient’s situation such as digital literacy, access to a digital device, internet connection and data, and a private environment
3)	Can prepare a telepsychiatry consultation that is culturally safe. These can include asking about cultural and religious protocols (e.g. karakia and prayer), involving whānau/family or cultural support, and arranging an interpreter
4)	Communicates effectively in a culturally safe manner during a telepsychiatry consultation
5)	Can work effectively with an interpreter during a telepsychiatry consultation
16)	Can build rapport and trust and show empathy with a patient despite the challenges associated with telepsychiatry, particularly at the first assessment
Domain 2: Infrastructure and technology	
6)	Can set up a telepsychiatry consultation and utilise various functionalities of telehealth platforms or videoconference programmes
8)	Can address technological problems and issues that may arise for a patient during a telepsychiatry consultation, including communication issues due to hardware issues or disruptions to the internet connection by arranging alternate consultation or contact options
9)	Manages practical engagement and technical issues that may arise during a complex consultation such as when multiple people are logged into the platform or present in the room with the psychiatrist or the patient
17)	Works effectively with clinical populations and situations that can most benefit from telepsychiatry
	For example, patients who present with anxiety/agoraphobia, live in rural and remote areas, have mobility/transport issue, are concerned about the stigma of attending a mental health clinic; family meeting with whanau/family members who are not living locally; involving a multidisciplinary team based in different localities; and continuity of care during a pandemic
Domain 3: Professional development and ethics	
10)	Applies the College’s professional practice guidelines and other relevant guidelines (e.g. Medical Council of New Zealand statement on telehealth) when practising telepsychiatry
11)	Engages in continuing professional development including peer review, reflective practice, obtaining feedback from telepsychiatry users, and completing training in telepsychiatry
13)	Obtains informed consent from a patient to use telepsychiatry, including explaining digital security, confidentiality and potential privacy issues that are specifically related to telepsychiatry (e.g. interviewed in their home, unwanted people in the room, recording a session)
15)	Is aware of and addresses specific work-life balance issues that arise from extensive use of telepsychiatry (e.g. working from home, productivity, online fatigue, peer support)
Domain 4: Clinical practice	
12)	Presents professionally during a telepsychiatry consultation
14)	Uses telepsychiatry effectively within a multi-disciplinary team setting and when communicating with other health professionals and health organisations. This can include managing team dynamics and providing support to other team members
18)	Recognises the relative contraindications and limitations of using telepsychiatry in certain clinical populations and situations
	For example, patients who present with significant risk and safety issues; older people with hearing and visual impairment; patients who are emotionally dysregulated or psychologically distressed; complex family/whanau dynamics; patients who are thought disordered or presented with psychosis such as persecutory delusions; performing a cognitive assessment or physical examination; completing rating scales; and following cultural protocols
20)	Complies with the legislative and documentation requirements when using telepsychiatry for assessment under the mental health act

**Table 5.** Internal consistencies of the new factor structure.

Competency domains	Self-rated competence α	Importance α
1. Cultural safety	0.86	0.85
2. Infrastructure and technology	0.82	0.82
3. Professional development and ethics	0.83	0.81
4. Clinical practice	0.68	0.75

## Discussion

Existing research in Aotearoa highlights the need to inform culturally safe telepsychiatry practice^9,10,12^ and better support and upskill mental health clinicians in telepsychiatry practice.^10,11^ Our study identified perceived gaps and opportunities to advance telepsychiatry practice in Aotearoa. Utilising a mixed-methods approach, the preliminary competency domains of ‘Cultural Safety’, ‘Infrastructure and Technology’, ‘Professionalism and Practice’, ‘Special Populations and Clinical situations’ were transformed to the four final domains of ‘Cultural Safety’, ‘Infrastructure and Technology’, ‘Professional Development and Ethics’, and ‘Clinical Practice’. This study has resulted in a culturally responsive framework to guide future development of education and training for effective delivery of telepsychiatry.

Relevant telehealth guidelines promoting best-practice for psychiatrists in Aotearoa include the Medical Council of New Zealand telehealth statement and the RANZCP professional practice guideline.^14,16^ These guidelines do not specify learning objectives or educational standards which our competency framework aims to address. Our competency domains could be incorporated into psychiatry registrar education such as a RANZCP Entrustable Professional Activity and inform relevant learning modules for psychiatrists working in both public and private settings.

Participants in our study indicated a lack of confidence in applying cultural safety guidelines. This may be in part due to cohort effect, coupled with a lack of existing guidelines regarding cultural safety in the telepsychiatry setting rather than awareness. Previous research has shown psychiatrists and trainees are mindful of the need to consider preparation and delivery of cultural practices prior to a telepsychiatry session, including *whānau* (family) and cultural liaison supports during sessions, and ease of continuing practice such as *karakia* (prayers or incantations).^12^ The lack of confidence in cultural safety may also be attributed to a lack of resourcing, including culturally appropriate environments and barriers for clients in accessing technology.^12^ Additionally, telehealth can be less conducive to engagement and *whakawhanaungatanga* (the process of establishing relationships)^12^ due to the value that Māori place on physical connectedness, as reported by Māori clinicians.^10^

Participant self-rated competence in working effectively with interpreters needs further exploration to understand the challenges. Using interpreters in telepsychiatry has previously been identified by psychiatrists and trainees as useful for Pacific peoples and Asian clients,^12^ highlighting the need for the development of protocols for their utilisation. Our finding that psychiatrists and trainees lacked self-rated competency in addressing technology problems mirrors the experience of mental health clients in Aotearoa, who described a need for mental health clinicians to improve telehealth literacy.^11^

The low response rate in this study could limit generalisability to the Aotearoa psychiatry community and there was potential for the participants to have biases towards preferring telepsychiatry.^17^ Despite this, the final four competency domains, based on the ratings of the importance of providing a culturally responsive telepsychiatry service have relatively good internal consistency, suggesting our framework is suitable for guiding telepsychiatry competency. A second limitation is that our telepsychiatry competency framework was derived in the Aotearoa setting and may not be applicable for other countries. Other countries may adapt this framework to meet the cultural needs in their settings, or they could use a similar methodology to survey psychiatrists’ and trainees’ perceptions of their competencies and their opinions on the importance of these competencies when providing a culturally responsive telepsychiatry service and to refine the framework using PCF analysis.

In conclusion, we extended the previous qualitative work on telepsychiatry competency by using quantitative methods to develop a telepsychiatry competency framework with four competency domains and 18 competency statements. This framework could be used in future work on developing educational curricula and training resources to improve the practice of telepsychiatry in Aotearoa.

## Supplemental Material

Supplemental Material - Developing a culturally informed telepsychiatry competency framework for aotearoa New Zealand: A cross-sectional survey and factor analysis Supplemental Material for Developing a culturally informed telepsychiatry competency framework for aotearoa New Zealand: A cross-sectional survey and factor analysis by Alisha Vara, Yan Chen, Etuini Ma’u, Rodrigo Ramalho, Mark Lawrence and Gary Cheung in Australasian Psychiatry.

## Data Availability

Data can be accessed upon establishing a data-sharing agreement and obtaining approval from the appropriate ethics committee.*
