# Supplementary material for: Developing a culturally informed telepsychiatry competency framework for aotearoa New Zealand: A cross-sectional survey and factor analysis
Source: Australas Psychiatry. 2025 May 28;33(6):902–8. doi: 10.1177/10398562251345313 (PMC12657665; doi:10.1177/10398562251345313)
Supplement: Supplemental Material - Developing a culturally informed telepsychiatry competency framework for aotearoa New Zealand: A cross-sectional survey and factor analysis [file sj-pdf-1-apy-10.1177_10398562251345313.pdf]

Supplementary material: The preliminary four competency domains and 20 competency statements

Domain 1: Cultural safety

NB: Domain 1 competency statements form the foundation for competency statements listed in the other 3 domains.

1) Applies official guidelines on cultural safety when practising telepsychiatry. For example, the New Zealand Medical Council's statement on cultural safety and He Ara Hauora Māori: A Pathway to Māori Health Equity.

2) Demonstrates an appreciation of inequity in accessing telepsychiatry in some communities and endeavours to accommodate these. For example, practical issues and wider social considerations of a patient's situation such as digital literacy, access to a digital device, internet connection and data, and a private environment.

3) Can prepare a telepsychiatry consultation that is culturally safe. These can include asking about cultural and religious protocols (e.g., karakia and prayer), involving whānau/family or cultural support, and arranging an interpreter.

4) Communicates effectively in a culturally safe manner during a telepsychiatry consultation.

5) Can work effectively with an interpreter during a telepsychiatry consultation.

Domain 2: Infrastructure and technology

6) Can set up a telepsychiatry consultation and utilise various functionalities of telehealth platforms or videoconference programmes.

7) Understands the breadth of clinician responsibilities when arranging a telepsychiatry consultation including both technological and clinical issues. These can include assisting a patient to set up for the consultation and the flexibility required to use a telehealth platform a patient prefers or has access to.

8) Can address technological problems and issues that may arise for a patient during a telepsychiatry consultation, including communication issues due to hardware issues or disruptions to the internet connection by arranging alternate consultation or contact options.

9) Manages practical engagement and technical issues that may arise during a complex consultation such as when multiple people are logged into the platform or present in the room with the psychiatrist or the patient.

Domain 3: Professionalism and practice

10) Applies the College's professional practice guidelines and other relevant guidelines (e.g. Medical Council of New Zealand statement on telehealth) when practising telepsychiatry.

11) Engages in continuing professional development including peer review, reflective practice, obtaining feedback from telepsychiatry users, and completing training in telepsychiatry.

12) Presents professionally during a telepsychiatry consultation.

13) Obtains informed consent from a patient to use telepsychiatry, including explaining digital security, confidentiality and potential privacy issues that are specifically related to telepsychiatry (e.g. interviewed in their home, unwanted people in the room, recording a session).

14) Uses telepsychiatry effectively within a multi-disciplinary team setting and when communicating with other health professionals and health organisations. This can include managing team dynamics and providing support to other team members.

15) Is aware of and addresses specific work-life balance issues that arise from extensive use of telepsychiatry (e.g. working from home, productivity, online fatigue, peer support).

Domain 4: Special populations and clinical situations

16) Can build rapport and trust and show empathy with a patient despite the challenges associated with telepsychiatry, particularly at the first assessment.

17) Works effectively with clinical populations and situations that can most benefit from telepsychiatry.

    For example, patients who present with anxiety/agoraphobia, live in rural and remote areas, have mobility/transport issue, are concerned about the stigma of attending a mental health clinic; family meeting with whanau/family members who are not living locally; involving a multidisciplinary team based in different localities; and continuity of care during a pandemic.

18) Recognises the relative contraindications and limitations of using telepsychiatry in certain clinical populations and situations.

For example, patients who present with significant risk and safety issues; older people with hearing and visual impairment; patients who are emotionally dysregulated or psychologically distressed; complex family/whanau dynamics; patients who are thought disordered or presented with psychosis such as persecutory delusions; performing a cognitive assessment or physical examination; completing rating scales; and following cultural protocols.

19) Can manage patient’s risks to self or others when they arise during a telepsychiatry consultation.

20) Complies with the legislative and documentation requirements when using telepsychiatry for assessment under the Mental Health Act.
